# Ocular Dominance Is Associated with the Ganglion Cell-Inner Plexiform Layer Thickness Profile in the Macula

**DOI:** 10.1371/journal.pone.0150035

**Published:** 2016-02-26

**Authors:** Jin A. Choi, Jung-Sub Kim, Hyun Jin Jeong, Jin Ah Lee, Chan Kee Park

**Affiliations:** 1 Department of Ophthalmology, St. Vincent’s Hospital, College of Medicine, The Catholic University of Korea, Suwon, Republic of Korea; 2 B & VIIT Eye Center, Seoul, Republic of Korea; 3 Department of Ophthalmology, Seoul St. Mary’s Hospital, College of Medicine, The Catholic University of Korea, Seoul, Republic of Korea; University of Florida, UNITED STATES

## Abstract

**Purpose:**

To investigate the characteristics of macular ganglion cell-inner plexiform layer (GCIPL) thickness profiles associated with ocular dominance.

**Setting:**

Private practice, Seoul, Republic of Korea.

**Design:**

Comparative case-control study.

**Methods:**

Both eyes of 199 participants with no ophthalmic abnormalities were included. Participants were imaged by spectral-domain optical coherence tomography, and underwent dominant eye testing using a hole-in-a-card test (sighting dominance) at the same visit. Macular GCIPL, as well as circumpapillary retinal nerve fiber layer (RNFL) thickness were compared for individual patients, according to ocular dominance.

**Results:**

Ocular dominance occurred predominantly in the right eye (right *vs*. left: 72.36 *vs*. 27.60%; *P* < 0.001). In the comparison of macular GCIPL thickness, the average (81.27±5.01 μm *vs*. 80.66±6.31 μm in dominant *vs*. non-dominant eyes), inferonasal (81.39±5.47μm *vs*. 80.33±6.82μm, and inferior sectors (77.95±6.05μm *vs*. 76.97±8.15μm) were significantly different between dominant and non-dominant eyes (*P* = 0.040, 0.005, and 0.032, respectively). Significant predictors of average GCIPL thickness were spherical equivalent (*β* = 1.37, *P*<0.001), astigmatic power (*β* = 1.44, *P* = 0.009), disc area (*β* = 3.90, *P* < 0.001), average RNFL thickness (*β* = 0.22, *P*<0.001), average cup-to-disc ratio (*β* = 5.74, *P* = 0.002), difference between the inferior and superior quadrant RNFL thicknesses (*β* = 0.08, *P* = 0.024), and ocular dominance (*β* = 2.10, *P* = 0.020). On multivariate regression analysis, ocular dominance was correlated with average GCIPL thickness after adjusting for potential confounders (*β* = 1.63, *P* = 0.048).

**Conclusions:**

Dominant eyes accompanied significantly thicker average macular GCIPL. This information suggests that macular GCIPL thickness may provide an indicator of the relative dominance of an eye.

## Introduction

Ocular dominance is defined as the relative input or preference of neurons throughout the visual system, especially primary visual cortex. It involves a suppression of the input from the non-dominant eye to avoid diplopia, and highly profound cases are associated with long term suppression, caused by ocular diseases such as amblyopia or strabismus [1]. The human retina exhibits semi-decussation, and the significance of ocular dominance differs from hemispheric laterality. However, the majority of visual input from one eye decussates and is processed in the contralateral cerebral hemisphere. Functionally, stimulation of the dominant eye evokes larger response than that of the non-dominant eye in the primary visual cortex [2]. In structural aspect, it has been also shown that the ocular dominance is related with the structural asymmetry in occipital complex [3]. In this regards, the ocular dominance seems to play a role in cerebral lateralization, and it is manifested structurally and functionally.

In retina, various structural differences between amblyopic and non-amblyopic eyes have been reported [4–6], although many studies reported no difference in total macular thickness [7–10]. However, the possible macular structural difference according to the ocular dominance has not been a focus of previous studies.

Ganglion cells are the final output neurons of the human retina, extending from the inner retina to the lateral geniculate nucleus in the midbrain. In retina, most of the ganglion cells exist at higher eccentricities within the central retina, and its density declines sharply in the peripheral retina. In addition, the macula has characteristically low convergence of foveal cones onto individual ganglion cells to preserve high-resolution images [11]. Recently developed optical coherence tomography (OCT) devices have enabled us to evaluate the ganglion cells more precisely [12–14]. With this recent advancement in OCT technology, we can now determine the entire retinal ganglion cell structure, from the dendrite/soma (ganglion cell-inner plexiform layer; GCIPL) to the axon (circumpapillary retinal nerve fiber layer (RNFL)). In this regard, we hypothesized that macular GCIPL analysis may be appropriate for assessing the effect of ocular dominance on retinal morphology.

To investigate this, we determined the profile characteristics of the macular GCIPL and RNFL that are associated with ocular dominance.

## Patients and Methods

### Study samples

Data was collected retrospectively on all subjects with myopia, who received preoperative examination for refractive surgery between October 2013 and December 2013 at the B & VIIT Eye Center, Korea. This study was performed according to the tenets of the Declaration of Helsinki. The institutional review and ethics boards of Seoul St. Mary’s Hospital approved the study protocol. The patient records/information was anonymized and de-identified prior to analysis.

A review of the medical history and a full ophthalmic examination were done at the initial visit. The examination included best-corrected visual acuity (BCVA) and refraction measurements; intraocular pressure (IOP) measurement using Goldmann applanation tonometry; stereo disc photo and fundus photography with digital fundus cameras (CR-1 mark II; Cannon, Tokyo, Japan), spectral-domain OCT, and perimetry (24–2 Swedish Interactive Threshold Algorithm standard automated perimetry, Humphrey Field Analyzer II; Carl Zeiss Meditec).

Patients were included if they have a healthy optic nerve head without glaucomatous damage (*i*.*e*., no disc hemorrhages, thinning, or notching of neural rim). Eyes with a glaucomatous visual field (VF) defects were excluded. A glaucomatous VF defect was defined as the presence of a cluster ≥ 3 points on the pattern deviation plot with a probability of occurrence of less than 5% in the healthy population, 1 point with the probability of occurrence in less than 1% of the healthy population, glaucoma hemifield test results outside the reference limits, or a pattern standard deviation with P < 5% [15].

Eyes with concurrent disease were also excluded. Other exclusion criteria included: eyes with a BCVA of <20/20; an IOP >21 mmHg in either eye; an ambiguous dominance in the hole-in-a-card test; a closed or occludable angle; a history of intraocular or refractive surgery; severe ocular trauma; media opacity; evidence of diabetic retinopathy or other vitreoretinal disease; evidence of optic neuropathy in either eye.

### The dominant eye testing

To determine the dominant eye, we utilized the hole-in-a-card test [16]. First, the patient was asked to hold a card with a hole centered in the middle using both hands, and to view a 6 m target through the hole in the card. Then each eye was occluded alternately by the observer to establish which eye is aligned with the hole and the distant target. The selected eye was considered to be the dominant eye. The process was repeated. Secondly, the patient was asked to move the card towards their face without losing alignment with the fixation target, until the hole was over an eye. This was considered to be the dominant eye.

### OCT Imaging

All of the subjects underwent imaging by spectral-domain OCT (Cirrus HD-OCT; Carl Zeiss Meditec). An optic-disc scan (Optic Disc Cube 200 × 200 protocol) and a macular scan (Macular Cube 514 × 128 protocol) were acquired by the same operator on the same day. The circumpapillary scan allowed measurement of RNFL thickness, whereas the macular scan allowed determination of macular GCIPL thickness using the GCA algorithm. Detailed descriptions of the Cirrus HD-OCT macular GCIPL, RNFL, and optic nerve head algorithms have been presented elsewhere [12–14]. Only well-focused, well-centered images without eye movement, with signal strengths of 7/10 or greater, were selected. For RNFL thickness measurements, the average RNFL thickness and the RNFL thickness for each quadrant sector were determined for all of the patients. In addition, the difference of RNFL thickness between the inferior and superior quadrant for each individual was calculated and designated as the RNFL I-S difference. For the GCIPL thickness measurements, the average and sectoral (superior [S], superonasal [SN], inferonasal [IN], inferior [I], inferotemporal [IT], and superotemporal [ST]) parameters were analyzed.

### Data analysis

Shapiro—Wilk testing was performed to test the normality of the distribution of the variables. All of the eyes were divided into two groups according to their ocular dominance (dominant eyes *vs*. non-dominant eyes). A two-tailed paired *t*-test was used to compare the means between dominant eyes and non-dominant eyes.

The determinant factors of average GCIPL thickness were analyzed using the generalized estimating equation. The dependent variable was average GCIPL thickness. The independent variables were age, spherical equivalent, astigmatic power, corneal thickness, disc area, rim area, average cup-to disc ratio, average RNFL thickness, RNFL I-S difference, ocular dominance, and cluster sampling. For multiple linear regression analysis, we first adjusted for potential confounders, such as age, sex, spherical equivalent, and disc area (Model 1). Then we adjusted for confounders that showed significant differences (*P* < 0.05), according to the average GCIPL thickness (Model 2). Statistical analyses were performed using SPSS for Windows, version 20.0 (SPSS, Chicago, IL). *P* values < 0.05 indicated statistical significance.

## Results

A total of 398 eyes of 199 participants were analyzed. Their mean age was 25.68 ± 6.67 years, and 37.2% were men (all Koreans). The IOP at the initial visit was 15.9 ± 2.9 mmHg. The mean spherical equivalent was −3.9 ± 2.0 D, and the mean corneal thickness was 539.6 ± 32.6 μm. The optic disc area was 1.76 ± 0.38 mm^2^, and the average RNFL thickness was 94.24 ± 9.50 μm. Ocular dominance occurred predominantly in the right eye (right *vs*. left: 72.36 *vs*. 27.60%; *P* < 0.001). The main characteristics of ocular dominance are shown in Table 1. Dominant eyes had significantly thinner corneal thickness (539.2 ± 32.7 μm *vs*. 540.0 ± 32.6 μm, *P* = 0.014), less astigmatic power (-1.0 ± 0.8 D *vs*. -1.2 ± 0.8 D, *P* = 0.005), and a deeper anterior chamber depth (3.23 ± 0.27 mm *vs*. 3.21 ± 0.28 mm, *P* = 0.029), compared to non-dominant eyes.

**Table 1:** Inter-ocular comparisons of clinical characteristics according to ocular dominance.

	Dominant Eyes	Non-dominant Eyes	*P*
	n = 199	n = 199	
Clinical characteristics			
Initial IOP (mmHg)	15.9±2.9	15.8±2.9	0.513
Corneal thickness (μm)	539.2±32.7	540.0±32.6	**0.014**
SE (Diopter)	-4.0±2.0	-3.9±2.0	0.143
Astigmatic power (Diopter)	-1.0±0.8	-1.2±0.8	**0.005**
ACD (mm)	3.23± 0.27	3.21±0.28	**0.029**
ONH parameter			
Disc area (mm^2^)	1.75±0.38	1.77±0.38	0.562
Rim area (mm^2^)	1.28±0.19	1.27±0.26	0.629
Average C/D ratio	0.46±0.16	0.47±0.16	0.096
RNFL thickness			
Average (μm)	94.93±7.99	93.54±10.77	**0.007**
IQR (μm)	(90.0–101.0)	(89.0–100.0)	
Superior quadrant (μm)	116.55±15.76	118.36±15.99	**0.036**
IQR (μm)	(106.0–129.0)	(108.0–129.0)	
Temporal quadrant (μm)	81.08±16.26	78.11±15.84	**<0.001**
IQR (μm)	(70.0–88.5)	(67.5–87.0)	
Inferior quadrant (μm)	118.81±16.59	117.85±17.14	0.272
IQR (μm)	(110.0–130.0)	(107.0–129.0)	
Nasal quadrant (μm)	63.45±10.69	61.53±9.71	**0.008**
IQR (μm)	(56.0–70.0)	(55.0–67.0)	
I-S difference	2.25±17.08	-0.51±18.64	**0.038**

Values are mean ± SD. IOP, intraocular pressure in mmHg; SE, spherical equivalent in diopter; ACD, anterior chamber depth in mm; C/D ratio, cup to disc ratio; RNFL, circumpapillary retinal nerve fiber layer thickness; IQR, interquartile range; I-S difference, inferior quadrant minus superior quadrant RNFL thickness. Means that significantly differed between each eye are in bold (*p*<0.05, paired t-test).

In the comparison of RNFL thickness, dominant eyes had significantly thicker average (mean [SD], 94.93μm [8.00 μm] in dominant eyes *vs*. 93.54 μm [10.77 μm] in non-dominant eyes; difference, 1.39μm; 95% confidence interval (CI), 0.383 to 2.39), temporal (81.08μm [16.26 μm] *vs*. 78.11 μm [15.84 μm]; 2.97μm; 95%CI, 1.45 to 4.49), and nasal RNFL thickness (63.45μm [10.69 μm] *vs*. 61.53 μm [9.71 μm]; 1.92μm; 95%CI, 0.51 to 3.32) (*P* = 0.007, <0.001, and 0.008, respectively), whereas non-dominant eyes accompanied significantly thicker superior RNFL thickness compared to dominant eyes (mean [SD], 116.56μm [15.76 μm] in dominant eyes *vs*. 118.36 μm [15.99 μm] in non-dominant eyes; difference, -1.81μm; 95%CI, -3.48 to -0.12 μm) (*P* = 0.036).

In the comparison of macular GCIPL thickness, the average (mean [SD], 81.27μm [5.01 μm] in dominant eyes *vs*. 80.66 μm [6.31 μm] in non-dominant eyes; difference, 0.61μm; 95%CI, 0.03 to 1.19μm), inferonasal (81.39μm [5.47μm] *vs*. 80.33μm [6.82μm]; 1.06μm; 95%CI, 0.32 to 1.79μm), and inferior sectors (77.95μm [6.05μm] *vs*. 76.97μm [8.15μm]; 0.97μm; 95%CI, 0.08 to 1.86μm) were significantly different between dominant and non-dominant eyes (*P* = 0.040, 0.005, and 0.032, respectively, Table 2). The average inferior sector was significantly thicker in dominant eyes (80.14μm [5.31μm] *vs*. 79.26μm [6.93μm]; 0.89μm; 95%CI, 0.19 to 1.58μm; *P* = 0.013).

**Table 2:** Inter-ocular comparisons of the macular ganglion cell-inner plexiform layer (GCIPL) according to ocular dominance.

	Dominant Eyes	Non-dominant Eyes	*P*
	n = 199	n = 199	
GCIPL parameter			
Average (μm)	81.27±5.01	80.67±6.31	**0.040**
IQR (μm)	(78.0–84.8)	(77.2–84.0)	
Minimal (μm)	78.26±7.80	77.87±9.22	0.513
IQR (μm)	(76.0–83.0)	(76.0–82.0)	
Superotemporal (μm)	80.86±5.26	80.46±6.48	0.284
Superior (μm)	82.61±5.63	82.44±6.79	0.641
Superonasal (μm)	83.79±6.35	83.47±6.80	0.441
Inferonasal (μm)	81.38±5.47	80.33±6.82	**0.005**
Inferior (μm)	77.94±6.05	76.97±8.15	**0.032**
Inferotemporal (μm)	81.09±5.45	80.47±7.01	0.086
Average superior sector (μm)	82.42±5.20	82.12±6.34	0.372
Average inferior sector (μm)	80.14±5.31	79.25±6.93	**0.013**

Values are mean ± SD. GCIPL, ganglion cell-inner plexiform layer; IQR, interquartile range. Means that significantly differed between each eye are in bold (*p*<0.05, paired *t*-test).

The average GCIPL thickness was associated with ocular dominance (*β* = 2.10, *P* = 0.020), spherical equivalent (*β* = 1.37, *P* < 0.001), astigmatic power (*β* = 1.44, *P* = 0.009), disc area (*β* = 3.90, *P* < 0.001), average RNFL thickness (*β* = 0.22, *P* < 0.001), average cup-to-disc ratio (*β* = 5.74, *P* = 0.002), and RNFL I-S difference (*β* = 0.08, *P* = 0.024), after adjusting for cluster sampling effects (Table 3).

**Table 3:** Univariate linear regression analysis of demographics and clinical variables: effect on average macular ganglion cell-inner plexiform layer thickness.

	Regression coefficient	Standard Error	95% Confidence interval	*P*
Age	0.081	0.062	-0.041, 0.204	0.194
Sex	-0.560	0.841	-2.209, 1.089	0.505
Spherical equivalent	1.370	1.809	1.016, 1.725	**<0.001**
Cylinder	1.445	0.552	0.363, 2.527	**0.009**
Corneal thickness	-0.006	0.012	-0.030, 0.019	0.652
Disc area	3.904	0.998	1.947, 5.860	**<0.001**
Rim area	2.808	1.742	-0.607, 6.224	0.107
Average RNFL thickness	0.224	0.028	0.169, 0.280	**<0.001**
Average CD ratio	5.741	1.849	2.117, 9.364	**0.002**
RNFL I-S difference	0.082	0.024	0.035, 0.129	**0.001**
Ocular dominance^a^	2.101	0.899	0.337, 3.864	**0.020**

^a^ Non-dominant eyes were used as the reference group. Results are adjusted for cluster sampling effects.

Finally, ocular dominance was significantly associated with the average GCIPL thickness after adjustment for age, sex, spherical equivalent, disc area, and cluster sampling effects (*P* = 0.016, Model 1), which was maintained after adjustment for confounders that showed significant differences according to the average GCIPL thickness (*P* = 0.048, Model 2, Table 4).

**Table 4:** Association between ocular dominance and average macular ganglion cell-inner plexiform layer thickness.

	Model 1			Model 2		
	Regression coefficient (95% CI)	Standard Error	*p*	Regression coefficient (95% CI)	Standard Error	*p*
Ocular dominance^a^	1.941 (0.366, 3.512)	0.804	**0.016**	1.626 (0.011, 3.241)	0.824	**0.048**

CI; confidence interval.

^a^ Non-dominant eyes were used as the reference group. Model 1: adjusted for age, sex, spherical equivalent, disc area, and cluster sampling effects. Model 2: adjusted for age, spherical equivalent, cylinder, disc area, average RNFL thickness, average cup-to disc ratio, RNFL I-S difference, and cluster sampling effects.

## Discussion

**Fig 1:**
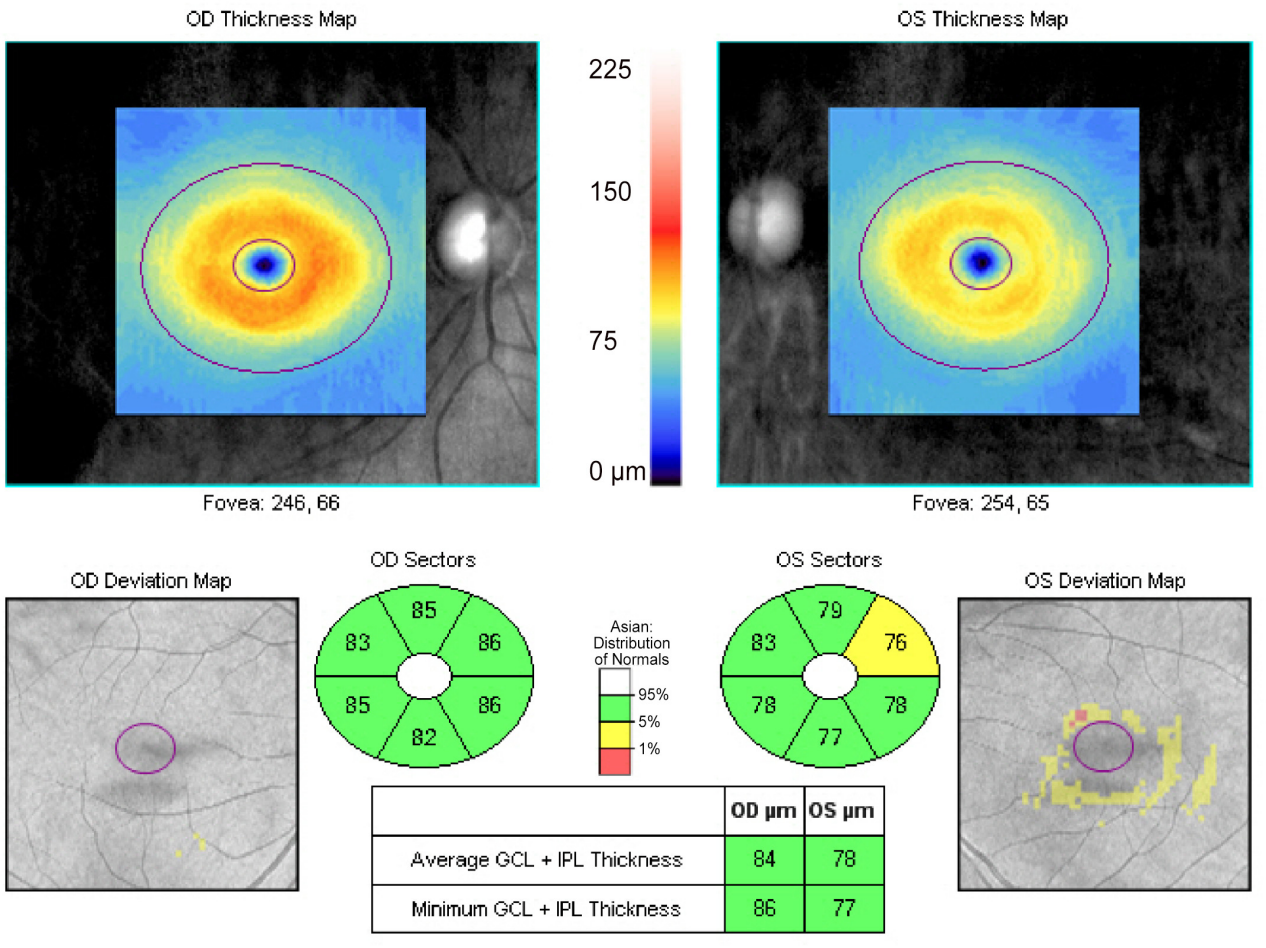
Representative case showing the characteristic of ganglion cell-inner plexiform layer (GCIPL) associated with ocular dominance. Images from a 36-year-old man with ocular dominance in his right eye. The average GCIPL thickness (84 μm) of the dominant eye was greater than that (78 μm) in the non-dominant eye. The average inferior GCIPL thickness of the dominant eye (84 μm) was also greater than that (78 μm) in the non-dominant eye.

In this study, we found that the circumpapillary RNFL and macular GCIPL distributions differed between dominant and non-dominant eyes. Dominant eyes had a significantly thicker average, temporal, and nasal RNFL thickness, whereas non-dominant eyes had thicker superior RNFL thickness compared to dominant eyes (Table 1). Finally, the average GCIPL of dominant eyes was significantly thicker than that of non-dominant eyes after controlling for other potential confounding factors, including age, spherical equivalent, astigmatic power, disc area, average cup-to disc ratio, average RNFL thickness, and RNFL I-S difference (Table 4). Representative case is shown in Fig 1. To the best of our knowledge, we are not aware of previous studies demonstrating the macular GCIPL characteristics associated with ocular dominance.

Consistent with former reports, ocular dominance occurred mostly in the right eyes (72%) [17,18]. Dominant eyes exhibited myopic tendencies, with significantly less astigmatic power (*P* = 0.005) compared to non-dominant eyes. This is consistent with the study of Chia et al [19], who investigated the effect of ocular dominance on refractive error in children in Singapore. However, spherical equivalent were not significantly different between dominant and non-dominant eyes.

There has not been a consensus regarding the retinal structural difference between amblyopic eyes and normal fellow eyes. Several studies have reported no difference in total macular thickness [7–10]. However, Repka et al [6]. demonstrated that amblyopic eyes tended to accompany thinner RNFL thickness, compared to contralateral sound eyes. Dickmann et al [4]. also showed that amblyopic eyes in a strabismic group showed significantly thinner macular thickness compared to fellow eyes. On the contrary, in the population-based Sydney Childhood Eye Study, children of 6 and 12 years of age had increased central macular thickness in eyes with amblyopia [5]. However, in their study, the inner macular ring (outer radius: 1.5 mm) was thinner in amblyopic eyes compared with sound eyes. In our study, macular GCIPL, which covers the central macula, excluding the central foveal region, was significantly thicker in dominant eyes compared to non-dominant eyes (Table 4). In this regard, it seems that ocular dominance affects the distribution of retinal signal-transduction cells (*i*.*e*., cone, rod, bipolar, and ganglion cells).

The mechanism regarding structural differences in the macula, with respect to ocular dominance, is not clear. One hypothesis is that the extent of reduction in retinal ganglion cells during normal postnatal development may differ according to the retinal region that each eye prefers [20]. Particularly in amblyopic eyes, the normal postnatal reduction of retinal ganglion cells may be inhibited to a greater degree. It was hypothesized that the arrest of normal postnatal reduction of retinal ganglion cells affects the normal maturation of the macula [5].

There is evidence that changes in the visual function accompany the changes in the relevant structures. It is well known that the retinal structural change in RNFL thickness is closely correlated with the visual functional change in glaucoma [21]. Recently, Gipponi et al. reported that RNFL thickness was decreased in migraine patients compared with normal patients [22]. As well as the loss of the structure, the asymmetry of the relevant structures also seems to reflect the functional changes in vision. In the study of Jensen et al. [3], ocular dominance was related to the structural asymmetry of cortical visual areas. In this regard, the differences of the average GCIPL thickness between the dominant *vs*. non-dominant eyes seem to contribute to the asymmetry between eyes, which functionally affect the ocular dominance.

Mwanza et al [12]., reported that the average GCIPL thickness was significantly associated with the average RNFL thickness, age, axial length, and male sex. Similarly, we identified spherical equivalent, astigmatic power, disc area, average cup-to-disc ratio, average RNFL thickness, and RNFL I-S difference, as significant predictors of normal GCIPL thickness, in addition to ocular dominance (Table 3). In their study, they reported there were no significant differences in average GCIPL thickness between right and left eyes. In a previous study, dominant eyes were accompanied by a thicker inferior RNFL and thinner superior RNFL, compared to non-dominant eyes [23]. Consistent with RNFL, the macular GCIPL at the inferior sector (particularly at inferonasal and inferior sectors) was thicker in dominant eyes (Table 2). It seems that ocular dominance is also related to the vertical distribution of the ganglion cell layer, and the preferred visual field for the eye with sighting dominance may be the superior hemifield that corresponds to the inferior retina, reflected in the RNFL profile in dominant eyes.

The differences of the average GCIPL thickness were relatively small between dominant eyes and non-dominant eyes in this study. Actually, the average GCIPL thickness is very symmetric between eyes, as shown in the former study, reporting no significant differences of the average GCIPL thickness between right and left eyes [12]. Another possible reason for the small differences between dominant and non-dominant eyes may be related to the fact that the average GCIPL thickness cannot reflect the regional variations in macula. Furthermore, in this study, the dominant and non-dominant eyes in healthy subjects were compared, rather than highly profound cases such as amblyopia or strabismus. This may explain the decreased structural differences between eyes.

Several limitations of this study should be acknowledged. First, the study participants were all Korean, and were relatively young with moderate myopia. To compensate for this, potential confounding factors, including spherical equivalent, were adjusted. We observed that the average GCIPL thickness was significantly associated with ocular dominance; however, a relatively small number of hyperopic or emmetropic eyes can lead to bias. In this study, only the sighting dominance test was used to determine ocular dominance, due to the simplicity of the test; however, determination of dominant eye is dependent on the test used, showing relatively poor intra-individual agreement between tests. Further studies using alternative technique such as +1.5D blur test for sensory dominance are warranted.

In conclusion, we found a small, but significant difference in macular GCIPL profile between dominant and non-dominant eyes. This information suggests that ganglion cell distribution in the macular area differs according to ocular dominance. The variation in the thickness of GCIPL profile in macula may be used as an indicator of the relative dominance of an eye, providing the evidence determining the dominant eye for refractive surgeons.

The English in this document has been checked by at least two professional editors, both native speakers of English. For a certificate, please see: http://www.textcheck.com/certificate/VCW3X0
